# L’ostéome ostéoïde de l’extrémité supérieure du radius: localisation rare et revue de la littérature (à propos d’un cas)

**DOI:** 10.11604/pamj.2022.41.313.17878

**Published:** 2022-04-18

**Authors:** Jawad Amghar, Mohammed Benhammou, Omar Agoumi, Abdelkrim Daoudi

**Affiliations:** 1 Service de Traumatologie Orthopédique A, Centre Hospitalier Universitaire Mohammed VI, Faculté de Médecine et de Pharmacie, Université Mohammed I, Oujda, Maroc

**Keywords:** Ostéome ostéoïde, extrémité supérieure du radius, exérèse, cas clinique, Osteoid osteoma, upper extremity of the radius, resection

## Abstract

L´ostéome ostéoïde est une tumeur osseuse bénigne, mais douloureuse et dont le traitement consiste en l´exérèse chirurgicale totale. Les auteurs rapportent le cas d´un jeune patient présentant un ostéome ostéoïde de l´extrémité supérieure du radius avec une revue de la littérature.

## Abstract (en)

Osteoid osteoma is a benign but painful bone tumor whose treatment is based on complete surgical resection. We here report the case of a young patient with osteoid osteoma of the upper extremity of the radius through a literature review.

## Introduction

L´ostéome ostéoïde est une tumeur osseuse bénigne douloureuse et pouvant se compliquer d´atteintes articulaires. Il affecte préférentiellement l´adolescent et le jeune adulte de sexe masculin. Il peut toucher tous les os, avec une prédominance pour les os longs. L´analyse anatomopathologique montre un nidus central hyper vascularisé, toujours inférieur à 2 cm, avec sclérose périphérique. Le traitement de référence est la chirurgie à ciel ouvert (résection en bloc du nidus) [1]. Nous rapportons, dans ce travail le cas d´un jeune patient présentant un ostéome ostéoïde de l´extrémité supérieure du radius avec une revue de la littérature.

## Patient et observation

**Information de la patiente:** monsieur BA 19 ans sans antécédents pathologiques (pas de notion de traumatisme), se plaignait de l´apparition au niveau du tiers supérieur de la face antéro-externe du bras gauche d´une masse douloureuse entrainant chez lui une impotence fonctionnelle partielle évoluant depuis 3 ans.

**Figure 1:**
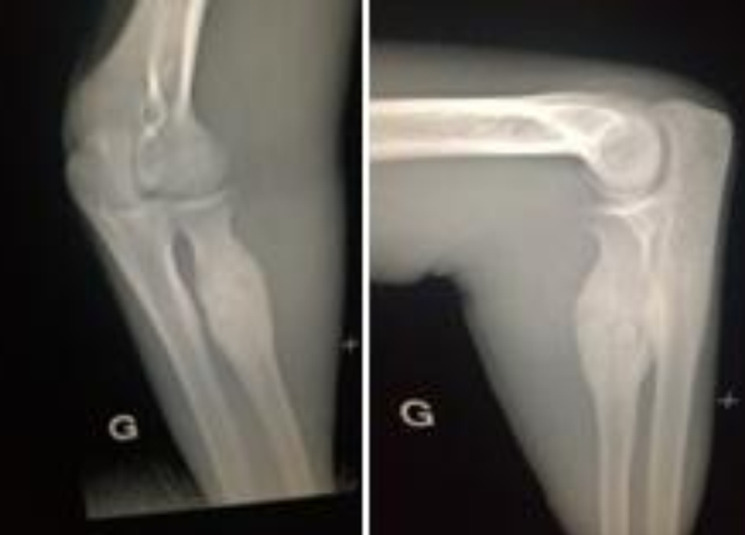
radiographie de face et de profil montrant l´image en cocarde sur l´extrémité supérieure du radius côté gauche

**Résultats cliniques:** à l´examen la masse faisait deux centimètres (2 cm) de grand axe, fixe par rapport au deux plans superficiel et profond, douloureuse à la palpation; pas de circulation veineuse collatérale ni de signes inflammatoires en regard. La mobilité articulaire: flexion, extension normales pour une prono-supination limité. Les aires ganglionnaires étaient libres et on n´avait pas noté de déficits sensitivo-moteurs. L´utilisation des anti-inflammatoires non stéroïdiens et des salicylées avait permis une diminution de la douleur pendant les 3 premiers mois puis on avait assisté à une recrudescence de la douleur malgré le traitement instauré. La douleur rapportée par le patient n´avait pas d´horaire particulier. L´imagerie radiologique avait objectivé un épaississement cortical centré par une lacune centimétrique (image en cocarde) évoquant un ostéome ostéoïde (Figure 1).

**Figure 2:**
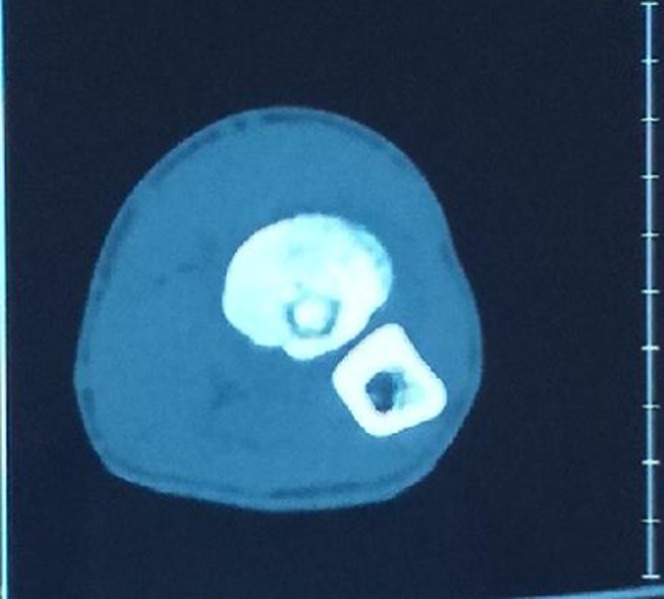
coupe axiale tomodensitométrique du coude gauche montrant la niche

**Figure 3:**
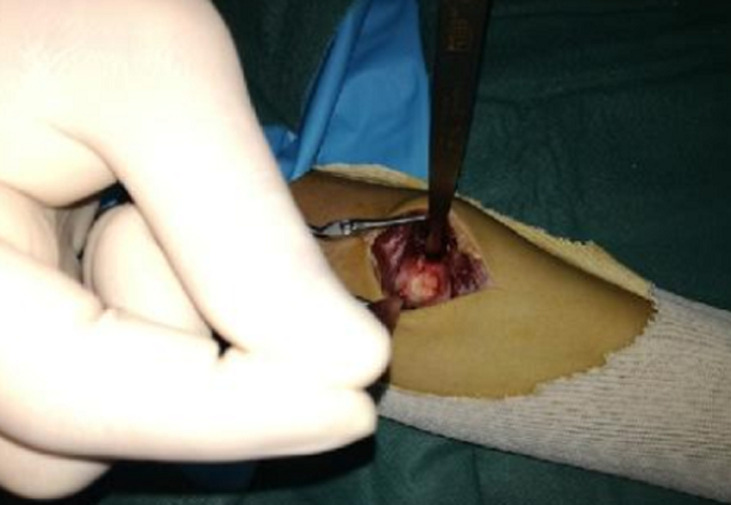
image préopératoire montrant le nidus

**Figure 4:**
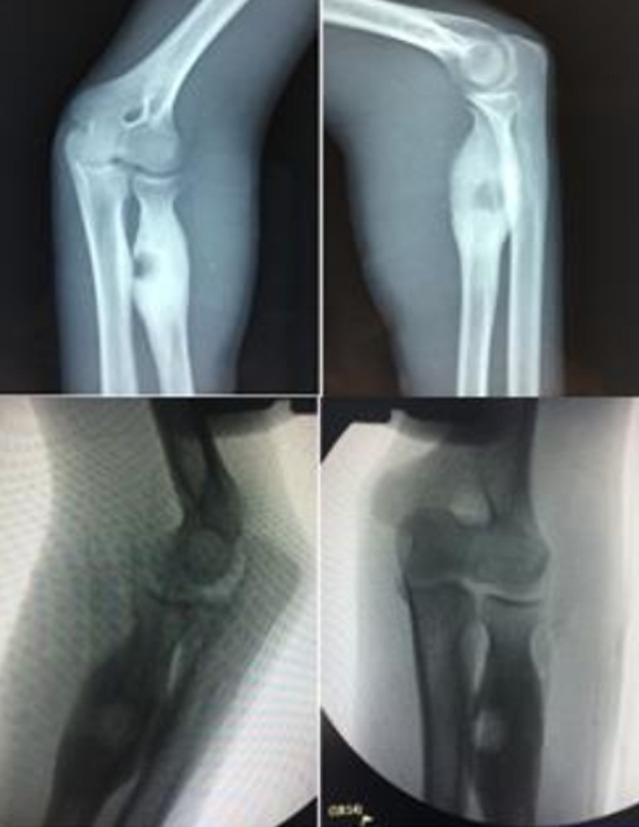
contrôle radiologique et scopique du coude gauche de face et de profil après exérèse

**Démarche diagnostique:** l´examen tomodensitométrique (Figure 2) du coude avait objectivé au niveau de l´extrémité supérieur du radius une lésion ostéolytique de taille inférieure à 1 cm bien limitée entourée d´une importante réaction ostéosclérose compatible avec un ostéome ostéoïde. Une intervention chirurgicale (Figure 3) avait permis de repérer la lésion suggestive de l´ostéome ostéoïde à l´aide de l´amplificateur de brillance ainsi que son exérèse (Figure 4). L´examen anatomopathologique avait confirmé le diagnostic de l´ostéome ostéoïde.

**Figure 5:**
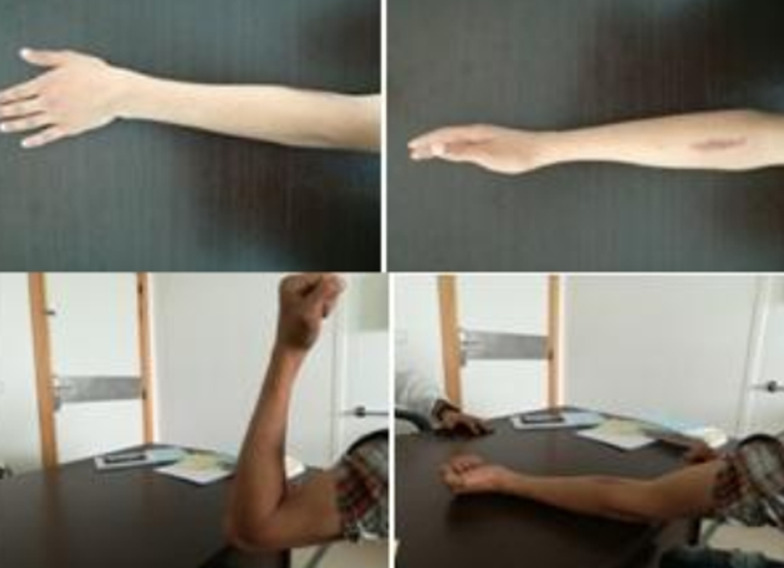
amplitude articulaire quatre mois après l´exérèse avec disparition totale de la douleur

**Intervention thérapeutique et suivi:** l´évolution post opératoire du patient était entièrement satisfaisante; tous les phénomènes douloureux avaient totalement disparu et la reprise totale de toute activité était possible au bout de trois mois (Figure 5).

## Discussion

L´ostéome ostéoïde est une tumeur osseuse primitive bénigne fréquente. Il représente 2 à 3% de l´ensemble des tumeurs osseuses et 10 à 20% de l´ensemble des tumeurs osseuses bénignes [2,3]. L´extrémité supérieure du radius est une localisation rarement rapportée. Il se situe préférentiellement au niveau des os longs [4,5] avec une prédilection pour les membres inférieurs [6], notamment le tibia et le fémur. Peu d´article de la littérature rapporte une telle localisation 1% des cas [7]. Les manifestations cliniques de l´ostéome ostéoïde sont le plus souvent faites de douleurs nocturnes, insomniantes, calmées par la prise de salicylés [8]. De ce fait, l´ostéome ostéoïde de l´extrémité supérieure du radius, malgré sa rareté, devrait toujours être considéré comme un diagnostic différentiel avec l´ostéoblastome, l´abcès de Brodie, l´ostéomyélite chronique, les arthropathies: (arthrite, algodystrophie ou un processus infectieux) chez les jeunes patients se présentant avec une histoire douloureuse sans aucun antécédent de traumatisme [1,7]. Le diagnostic clinique, la scintigraphie osseuse [9], le scanner [2,3,8] et dans certains cas l´IRM [10] rendent le diagnostic quasiment certain avant la confirmation histologique.

Néanmoins, ce diagnostic peut rencontrer de multiples difficultés devant des localisations inhabituelles notamment au niveau de l´extrémité supérieure du radius. En présence de toute atypie, une biopsie devra être pratiquée [6,9]. Dans la littérature, pour le traitement de cette tumeur bénigne, bien qu´elle puisse involuer spontanément après des années, plusieurs techniques sont utilisées: 1) abord chirurgical avec l'exérèse osseuse en bloc [6] comme dans notre cas; 2) résection percutanée scano-guidée [11]; 3) alcoolisation percutanée: biopsie-résection percutanée par petites tréphines et sclérose par alcoolisation et destruction complète de la lesion [12].

## Conclusion

La localisation de l´ostéome ostéoïde au niveau de l´extrémité supérieure du radius est rare et son diagnostic est actuellement facilité par l´apport des techniques d´imagerie médicales. En cas de doute diagnostic, la tomodensitométrie représente l´examen le plus spécifique permettant le diagnostic positif. L´exérèse chirurgicale complète de la lésion permet le plus souvent la guérison totale et évite les récidives. Elle peut être obtenue par chirurgie classique à ciel ouvert ou par techniques plus modernes mini-invasives : résection percutanée scano-guidé.
